# Enlightening Gliotoxin Biological System in Agriculturally Relevant *Trichoderma* spp.

**DOI:** 10.3389/fmicb.2020.00200

**Published:** 2020-03-12

**Authors:** Daniela Bulgari, Lisa Fiorini, Alessandra Gianoncelli, Michela Bertuzzi, Emanuela Gobbi

**Affiliations:** ^1^Piattaforma di Microbiologia Agroalimentare ed Ambientale (Pi.Mi.A.A.), AgroFood Lab, Department of Molecular and Translational Medicine, University of Brescia, Brescia, Italy; ^2^Piattaforma di Proteomica, AgroFood Lab, Department of Molecular and Translational Medicine, University of Brescia, Brescia, Italy

**Keywords:** mycotoxin, biological control, self-protection, *Aspergillus*, secondary metabolism, epidithiodioxopiperazine

## Abstract

Gliotoxin (GT) is a dual fungal secondary metabolite (SM). It displays pleiotropic activities and possesses medicinal properties and biocontrol abilities but, unfortunately, has toxic properties in humans. Various *Trichoderma* species are used as fungal biological control agents (BCAs), as a sustainable alternative for crop protection worldwide. Among them is *Trichoderma virens*, a GT-producing fungus. Since no information was available on the genetically coded prerequisites for the production of GT in other *Trichoderma* spp., genome analyses were carried out in 10 *Trichoderma* spp. genomes. Moreover, a real-time PCR assay setup *ad hoc* and high-performance liquid chromatography (HPLC) analyses were employed to understand the GT-producing biological systems in *T. virens* GV29-8 (TvGv29-8) and *Trichoderma afroharzianum* T6776 (TaT6776), two relevant biocontrol fungi. The structure of the GT biosynthesis genes (GT-BG) is polymorphic, with two distinct types associated with the ability to produce GT. GliH, a key protein for GT synthesis, is absent in most of the *Trichoderma* GT biosynthetic pathways, which may be the reason for their inability to produce GT. The GT-BG are expressed in TvGv29-8 as expected, while they are silent in TaT6776. Interestingly, in the GT-non-producing TaT6776, only *gliA* (putative GT transporter) and *gtmA* (putative GT *S*-methyltransferase) were induced by exogenous GT, underlining the ability of this strain to reduce the deleterious effect of the toxin. This ability is confirmed by growth assays and by the detection of the bis-thiomethylated form of GT catalyzed by GtmA in the culture medium supplemented with GT. To the best of our knowledge, this is the first general description of the GT biological system in different *Trichoderma* spp. as far as the GT-BG content and organization is concerned and a preliminary insight into their functionality.

## Introduction

Gliotoxin (GT) is a sulfur-containing fungal secondary metabolite (SM) of the class of epidithiodioxopiperazine (ETP), characterized by an internal disulfide bridge (Gardiner and Howlett, 2005; Dolan et al., 2015). Because of the presence of this bridge, GT can perform a redox cycling between the oxidized and reduced forms producing reactive oxygen species (ROS) (Gardiner and Howlett, 2005; Schrettl et al., 2010) or disturb the intracellular redox homeostasis by interacting with the thiol groups of antioxidants such as glutathione (Scharf et al., 2016).

Gliotoxin is produced by a phylogenetically diverse range of fungal species including *Aspergillus fumigatus*, *Eurotium chevalieri*, *Trichoderma virens*, *Neosartorya pseudofischeri*, and some *Penicillium* and *Acremonium* spp. This ETP can be detected in natural substrates, in the soil, in the rhizosphere, or as a contaminant of silage and other food commodities associated with the presence of fungi like *A. fumigatus* (Scharf et al., 2016). Remarkably, GT is produced by >96% of both environmental and clinical isolates of *A. fumigatus*, the opportunistic human pathogen that causes important and fatal aspergillosis, where it represents a virulence factor (Sugui et al., 2007) by means of its pleiotropic immunosuppressive activity (Konstantinovas et al., 2017).

The 13 canonical GT biosynthesis genes (GT-BG) are organized in a cluster (gli) in *A. fumigatus* (among others, Dolan et al., 2015), as it frequently occurs for fungal SM genes (Keller, 2019). The gli cluster includes the biosynthetic genes, the Zn(II)_2_-Cys(6) binuclear cluster domain transcription factor *gliZ*, and two self-protection genes, *gliT* (a GT oxidoreductase mediating the oxidation of sulfhydryl groups to form the disulfide bridge) and *gliA* (the GT transporter). In addition to being regulated by *gliZ*, both *gliT*, and *gliA* are also differentially regulated by a yet unknown regulator (Gardiner and Howlett, 2005; Schrettl et al., 2010) and by the transcription factor *gipA* (Schoberle et al., 2014). The environmental stimuli that induce expression of the gli cluster are still uncharacterized (Scharf et al., 2016). However, GT generally influences the expression of the biosynthetic genes in a positive feedback loop (Cramer et al., 2006). In the presence of GT, *A. fumigatus* produces also the bis-thiomethylated form of GT (BmGT) catalyzed by the GT *S*-methyltransferase GtmA. Indirectly, this enzyme negatively regulates the GT biosynthesis by disrupting the positive feedback loop, and it is not primarily involved in the self-protection mechanism (Dolan et al., 2014).

Even though GT was first discovered in *T. virens* (also described in literature as *Gliocladium virens*) (Weindling, 1934), few studies have been carried out on GT presence in this species, whose isolates were classified as “Q” strains, if they produced GT and BmGT but not gliovirin or heptelidic acid, or “P” strains, if they produced gliovirin and heptelidic acid but neither GT nor BmGT (Howell et al., 1993).

The role of GT is still debated: in *A. fumigatus*, it not only is a virulence factor (Sugui et al., 2007), but it also shows a protective role against oxidative stress (Owens et al., 2014); in the biological control agent *T. virens*, GT is thought to be linked to antagonism (Kubicek et al., 2011).

The gli cluster in *T. virens* is known to comprise only 8 of the 13 genes reported in *A. fumigatus* with two putative *gliT* orthologs (TRIVIDRAFT_138628 and TRIVIDRAFT_31354) and one putative *gliZ* ortholog (TRIVIDRAFT_191964) located elsewhere in the genome (Mukherjee et al., 2012; Vargas et al., 2014). Besides these, to the best of our knowledge, the only other reports on GT-BG in *Trichoderma* spp. refer to *Trichoderma reesei* where either a truncated GT cluster with only 8 of the 13 *gli* genes (Mukherjee et al., 2012) or an ETP gene cluster of 12 genes (Patron et al., 2007) were described; importantly, both reports agreed on the absence of GT production by the *T. reesei* strains.

*Trichoderma* isolates are among the most frequently used microorganisms as active principles of commercial biofertilizers and biopesticides, yet nine species among which *T. longibrachiatum*, *T. atroviride*, *T. harzianum*, *T. reesei*, and *T. viride* are infrequent but emergent opportunistic human pathogens (Druzhinina et al., 2008; Sandoval-Denis et al., 2014; Sautour et al., 2018). Although biopesticides derived from naturally occurring microorganisms are generally considered to provide an environmentally benign pest control option, they may not be entirely free of hazards and consequently require an accurate risk assessment (Konstantinovas et al., 2017; Bulgari et al., 2019).

Although GT represents a virulence factor for *A. fumigatus*, few studies aimed to monitor the presence of GT or the *gli* biosynthesis genes (GT-BG) in *Trichoderma* spp.

In the present study, the GT-BG structure in six representative *Trichoderma* species was obtained, taking advantage of the fact that several genome sequences of *Trichoderma* species are now available, providing the opportunity to rapidly screen fungal genomes for the presence of the gli cluster and compare its organization between different species. Moreover, the GT biological system was characterized by high-performance liquid chromatography (HPLC) analyses in the agriculturally relevant strains *T. virens* Gv29-8 and *T. afroharzianum* T6776 and a useful real-time reverse transcription PCR (RT-PCR) assay was developed *ad hoc* to follow the expression of their *gli* genes encoding GliP, GliT, GliA, GliH, GipA, and GtmA in GT permissive conditions.

## Materials and Methods

### Strains and Growth Conditions

The experimental studies were carried out on the biocontrol agent *T. virens* Gv29-8, a GT-producing strain, and on the plant growth promoter *T. harzianum* T6776 (Fiorini et al., 2016), recently reclassified as *T. afroharzianum* T6776 (Kubicek et al., 2019). The fungal strains were routinely cultivated on potato dextrose agar (PDA) or potato dextrose broth (PDB) as specified.

### Bioinformatic Analyses of Gliotoxin Biosynthesis Genes

The fungal strains, whose genome sequences were downloaded from the National Center for Biotechnology (NCBI) for the *in silico* study, are presented in Table 1 with their related abbreviations and other information. *A. fumigatus* Af293 was used as a reference strain and its annotated genome as query. Different basic local alignment search tool (BLAST) analyses (blastn, tblastn, blastp, blastx) were performed using the Af293 gli cluster gene and protein sequences as a query to further assess content and arrangements of genes in TvGv29-8. Successively, the TvGv29-8 *gli* genes and protein sequences were also used as references to find the putative orthologous *gli* genes among *Trichoderma* spp. For the *gliH* search, the *AfgliH* described by Schrettl et al. (2010) was used instead of AFUA_5G10320, a gene annotated as *gliH* in the NCBI database. The highest scoring and covering hit for each gene was verified by the bidirectional best hits (BBH) approach against Af293 and the other *Trichoderma* genomes and eventually chosen, identified, and localized on the *Trichoderma* spp. genomes. Each gene orthology was then verified in the OrthoDB catalog of eukaryotic orthologs provided as an option in NCBI. To clearly distinguish between orthologous genes in *A. fumigatus*, *T. virens*, *T. afroharzianum*, *T. harzianum*, *T. reesei*, *T. gamsii*, and *T. longibrachiatum*, gene designations are preceded, respectively, by the prefix *Afgli*, *Tvgli*, *Tagli*, *Thgli*, *Trgli*, *Tggli*, and *Tlgli*. The *Afgli* genes and putative GT-BG of the analyzed *Trichoderma* strains are listed in Supplementary Table S1.

**TABLE 1:** Fungal strains used or whose genomes were used in this study.

Organism	Strain	Collection	NCBI Bioproject no.	Designation in this study
*Aspergillus fumigatus*	Af293	NCPF 7367, CBS 101355	PRJNA131	Af293
*Trichoderma virens*	Gv29-8	ATCC MYA-4894	PRJNA19983	TvGv29-8
	FT-333	National Chiayi University, Taiwan	PRJNA268050	TvFT333
*Trichoderma afro-harzianum*	T6776	University of Pisa, Italy	PRJNA252551	TaT6776
*Trichoderma harzianum*	B97	Biovitis, France	PRJNA357189	ThB97
	TR274	Universidade Fed. de Goias, Goiânia, Brazil	PRJNA397414	ThTR274
	CBS 226.95	CBS 226.95	PRJNA207867	Th226.95
*Trichoderma reesei*	RUT-C30	ATCC 56765	PRJNA207855	TrRUTC30
	QM6a	ATCC 13631	PRJNA15571	TrQM6a
*Trichoderma gamsii*	T6085	University of Pisa, Italy	PRJNA252048	TgT6085
*Trichoderma longibrachiatum*	SMF2	Shandong University, Jinan, China	PRJNA175761	TlSMF2

### Selection of Candidate Genes and Primers Design

To verify the correct identification and functionality of the gli clusters in TvGv29-8 and TaT6776, candidate reference genes for gene expression analyses were shortlisted. In detail, *gliP*, coding the key GT biosynthetic bimodular non-ribosomal peptide synthetase; *gliH*, unknown function; *gliT* and *gliA*, self-protection genes; *gliZ* and *gipA*, transcription factors; and *gtmA*, a gene belonging to a negative regulatory system, were chosen as the main representative genes for GT biosynthesis, self-protection, and regulation, respectively. Calmodulin (*TCal*) and secretion-associated Ras-related protein (*TSar*) genes were used as housekeeping genes for gene expression normalization. Real-time RT-PCR protocols were developed for all these genes. Each primer was designed in an intron flanking region. Specific primer pairs were designed using Primer3Plus. The gene names, amplification efficiency, correlation coefficient, amplicon length, and amplicon melting temperature are reported in Supplementary Table S2. The amplification efficiency of quantitative PCR (qPCR) was calculated only for the genes expressed in the absence and the presence of exogenous GT and ranged from 90 to 99.8%, and the *R*^2^ of the standard curve was 0.997 ± 0.001. Agarose gel electrophoresis and melting curve analyses (data not shown) showed a single temperature for each primer pair that was distinctly different from the non-specific genomic peak, confirming the specificity of the amplification.

### RNA Extraction and Real-Time PCR

TvGv29-8 and TaT6776 conidia were inoculated in PDB at a final concentration of 10^7^ conidia/ml and incubated at 23–25°C under illumination of 16 h light/8 h dark cycles, using daylight tubes 24 W/m^2^, 9,000 lx. After 27 h, fungal RNA was isolated and purified using the PureLink RNA purification kit (Ambion, Thermo Scientific). To avoid genomic DNA (gDNA) contaminations, the RNAs were treated with DNase I (Ambion, Thermo Scientific), and complementary DNA (cDNA) synthesis from messenger RNA (mRNA) (200 ng) was performed using cDNA first-strand synthesis kit (Thermo Fisher) with hexamer primer according to manufacturer’s instruction.

Reactions were performed using the Real-Time PCR PowerUp SYBR Green Master Mix kit (Applied Biosystems) in the ViiA7 Real-Time PCR system (Applied Biosystems). Each RT-PCR reaction was performed in three biological and technical replicates and a no-template control. To determine the specificity of the primer pairs used in the current study, 1.5% agarose gel electrophoresis and melt curve analyses were performed (Supplementary Table S2). To determine the PCR efficiency of each primer pair, a standard curve was generated using linear regression, and the Cq slope was calculated based on the Cq values for all dilutions (5 points, 10-fold dilutions from 50 to 50,000).

### Induction With Exogenous Gliotoxin

TvGv29-8 and TaT6776 were inoculated in PDB by spore suspension at a final concentration of 10^7^ conidia/ml and incubated at 23–25°C under illumination of 16 h light/8 h dark cycles, using daylight tubes 24 W/m^2^, 9,000 lx. After 24 h, exogenous GT (WVR International) or methanol (MeOH, solvent control) was added to the media at 5 μg/ml (final concentration) and incubated for 3 h under the same conditions. Mycelia were harvested through Miracloth filtration, washed with distilled water, and immediately frozen in liquid nitrogen and processed for gene expression analyses as described above. The culture filtrates were stored at −20°C until GT identification and quantification by HPLC analysis.

### GT and BmGT Production in Liquid Media

Conidia (1 × 10^6^ conidia) of TvGv29-8, TaT6776, and TrRUTC30 (as negative control) were inoculated into 25 ml Weindling modified medium (Wilhite and Straney, 1996) and cultured on a rotary shaker (150 rpm) at 25°C of 16 h light/8 h dark cycles, using daylight tubes 24 W/m^2^, 9,000 lx. To investigate the time course of GT production, aliquots of the culture filtrates were taken at incubation times 24, 48, 72, 96, and 162 h and analyzed by HPLC. Three replicates were performed for each fungus. For BmGT quantification, samples were taken at 27 h incubation time.

### Extraction and Estimation of Gliotoxin by HPLC

Culture filtrate aliquots were diluted with MilliQ water (1:1) and extracted three times with half volume of chloroform while shaking (150 rpm) for 10 min. The concentrated chloroform extracts were dried over sodium sulfate, and the solvent was removed by Rotavapor (37°C, vacuum); the residue was dissolved in 200 μl methanol. Only the samples induced with exogenous GT were furtherly diluted 1:10. All the samples were then subjected to HPLC analysis.

Ten microliters of each biological sample was analyzed in triplicate by reverse-phase HPLC with UV detection (RP-HPLC-UV) using a Dionex^TM^ UltiMate^TM^ 3000 (Thermo Fisher Scientific) equipped with an LPG-3400SD quaternary analytical pump, a WPS-3000SL analytical autosampler, a VWD-3100 UV–visible detector, a TCC-3000SD thermostatted column compartment, and an AFC-3000 automatic fraction collector. Chromatographic separation was performed using a Waters XSelect CSH C18 column (150 mm × 2.1 mm ID; particle size, 3.5 μm). The specific liquid chromatographic (LC) parameters were as follows: mobile phase (A) water, TFA (99.95:0.05 *v*/*v*); (B) acetonitrile, the mobile phase flow rate was 0.3 ml/min; gradient program, 10% B for 2 min, from 10 to 90% B in 18 min, 90% B for 1 min and then from 90 to 10% B in 1 min and re-equilibration to 10% B for 3 min. All analyses were performed at 30°C. The detection wavelength (*λ*) was set at 254 nm.

As reference standard GT (VWR International) and BmGT (Sigma-Aldrich Co.) were used. For GT quantitation, a calibration curve was generated using five different dilutions of the GT reference standard (550.00, 275.00, 55.00, 27.50, and 5.50 μg/ml) and similarly for BmGT quantitation, using five different dilutions of the BmGT reference standard (50.00, 25.00, 10.00, 5.00, 1.00 μg/ml). Ten microliters of each standard dilution was analyzed by RP-HPLC-UV as reported above. The calibration curve was plotted using the area under peak versus GT or BmGT standard reference concentrations (μg/ml) (Supplementary Figures S1A,B, respectively). Biological sample concentrations were calculated by determining their area under peak and comparing them to the area of the calibration curve.

### *Trichoderma* Multiwells Growth Assay With Exogenous GT

TvGv29-8 and TaT6776 conidia at a final concentration of 10^6^ conidia/ml were inoculated in PDB containing different concentrations of GT or MeOH (0, 5, 15, 30, 50 μg/ml) in 96-well plates. The optical density (620 nm) of the liquid cultures was read at least three times a day at regular intervals, using an ELISA plate reader.

## Results

### *In silico* Identification of the GT Biosynthesis Genes and Related Genes in *Trichoderma virens*

Three new putative orthologs – *TvgliJ*, *TvgliA*, and *TvgliH* encoding a dipeptidase, a transporter, and an unknown function protein, respectively – were identified in TvGv29-8, at other loci, outside of the previously described gli cluster on scaffold 47. The relative position and characteristics of the *Tvgli* genes are shown in Table 2 and Supplementary Table S1. In detail, *TvgliJ*, located on scaffold 51, encodes a protein with a 52.81% sequence identity to AfGliJ; *TvgliA*, situated on scaffold 4, codes for TvGliA with 45.13% sequence identity to AfGliA; and *TvgliH*, on scaffold 10, encodes TvGliH with 54.40% sequence identity to AfGliH. Interestingly, of the *TvgliT* orthologs previously described, TRIVIDRAFT_138628 was found beside *TvgliH*, while TRIVIDRAFT_31354 was located on the large scaffold 4 where *TvgliA* is also present. In addition, two related genes, *TvgtmA*, the GT thiomethyltransferase, and *TvgipA*, a C_2_H_2_ transcription factor, were identified on TRIVI scaffolds 77 and 5, respectively.

**TABLE 2:** Sequence similarity analyses of the *gli* gene orthologs in *T. virens*, *T. afroharzianum*, *T. reesei*, and *T. longibrachiatum*.

*T. virens* Gv 29-8				*T. afroharzianum* T6776				*T. reesei* RUT-C30				*T. reesei* QM6a				*T. longibrachiatum* SMF2			
*Gene*	Query	*E* value	Ident	*Gene*	Query	*E* value	Ident	*Gene*	Query	*E* value	Ident	*Gene*	Query	*E* value	Ident	*Gene*	Query	*E* value	Ident
*TvgliZ*	26%	1e-20	53%	*TagliZ*	–	–	–	*TrgliZ*	–	–	–	*TrgliZ*	–	–	–	*TlgliZ*	–	–	–
*TvgliI*	96%	1e-155	52%	*TagliI*	96%	8e-91	35%	*TrgliI*	96%	4e-108	39%	*TrgliI*	97%	1e-108	40%	*TlgliI*	96%	1e-88	34%
*TvgliJ*	99%	2e-149	53%	*TagliJ*	92%	2e-133	50%	*TrgliJ*	95%	3e-144	55%	*TrgliJ*	95%	3e-144	55%	*TlgliJ*	92%	2e-122	47%
*TvgliP*	95%	0.0	44%	*TagliP*	95%	0.0	35%	*TrgliP*	98%	0.0	40%	*TrgliP*	98%	0.0	40%	*TlgliP*	95%	0.0	38%
*TvgliC*	90%	8e-157	48%	*TagliC*	89%	9e-131	43%	*TrgliC*	96%	1e-155	46%	*TrgliC*	89%	9e-155	49%	*TlgliC*	87%	9e-151	48%
*TvgliM*	96%	5e-174	56%	*TagliM*	87%	1e-119	45%	*TrgliM*	85%	8e-122	47%	*TrgliM*	85%	8e-122	47%	*TlgliM*	77%	5e-120	51%
*TvgliG*	95%	5e-113	68%	*TagliG*	78%	8e-37	38%	*TrgliG*	82%	8e-55	43%	*TrgliG*	82%	8e-55	43%	*TlgliG*	78%	2e-41	41%
*TvgliK*	89%	7e-83	47%	*TagliK*	94%	2e-53	38%	*TrgliK*	94%	6e-66	40%	*TrgliK*	94%	6e-66	40%	*TlgliK*	95%	6e-44	44%
*TvgliA*	98%	1e-160	45%	TagliA*	26%	0.39	40%	TrgliA*	16%	0.90	23%	TrgliA*	16%	0.82	23%	TlgliA*	19%	1.4	23%
*TvgliN*	98%	4e-123	58%	*TagliN*	97%	2e-32	26%	*TrgliN*	97%	4e-32	27%	*TrgliN*	97%	4e-32	27%	*TlgliN*	96%	2e-27	26%
*TvgliF*	99%	0.0	61%	*TagliF*	39%	7e-18	27%	*TrgliF*	65%	6e-20	23%	*TrgliF*	88%	1e-58	27%	*TlgliF*	44%	1e-10	27%
				*TagliF*°	34%	1e-11	24%	*TrgliF*°	34%	1e-09	23%	*TrgliF*°	34%	2e-09	23%				
TvgliT*	86%	1e-115	58%	*TagliT*	93%	8e-42	33%	*TrgliT*	92%	1e-53	36%	*TrgliT*	92%	1e-53	36%	*TlgliT*	91%	2e-44	33%
TvgliT**	87%	3e-107	52%																
*TvgliH*	96%	5e-68	54%	*TagliH*	–	–	–	*TrgliH*	–	–	–	*TrgliH*	–	–	–	*TlgliH*	–	–	–
*TvgipA*	60%	5e-44	51%	*TagipA*	97%	4e-48	36%	*TrgipA*	48%	4e-44	51%	*TrgipA*	48%	4e-44	51%	*TlgipA*	35%	5e-30	41%
*TvgtmA*	87%	7e-43	32%	*TagtmA*	87%	5e-41	31%	*TrgtmA*	26%	3e-04	34%	*TrgtmA*	92%	8e-41	30%	*TlgtmA*	90%	8–16	31%

The results are indicated as Query coverage (Query), BLAST *E* value (*E* value), and identity percentage (Ident) of deduced protein sequences with *A. fumigatus* gli proteins as references. gliA*, ABC transporters; putative orthologs present in *T. afroharzianum*, *T. reesei*, and *T. longibrachiatum*; gliT*, putative ortholog corresponding to TRIVIDRAFT_138628; gliT**, putative ortholog corresponding to TRIVIDRAFT_31354*; gliF*°, putative 2° ortholog present in *T. afroharzianum* and *T. reesi*.; – not detected.

**FIGURE 1:**
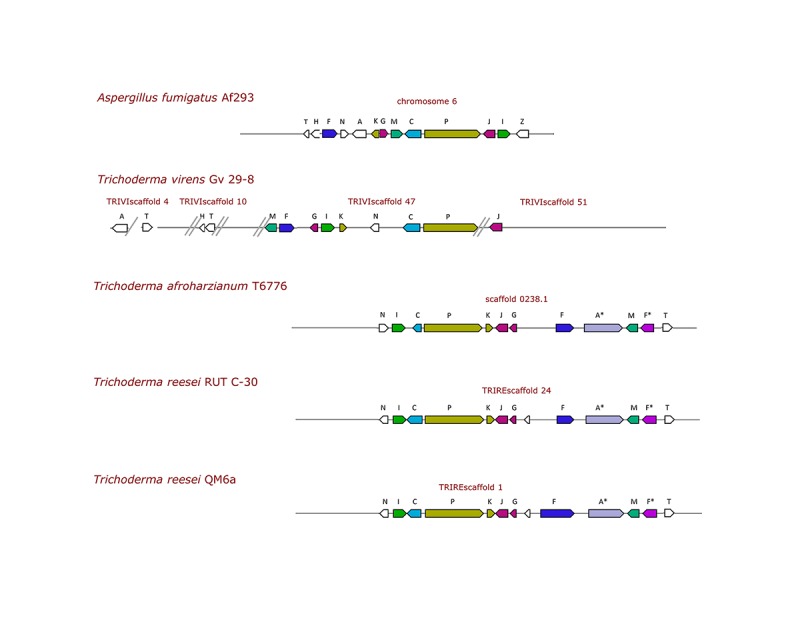
Schematic view of the gliotoxin biosynthesis genes (GT-BG) in *A. fumigatus* and representative *Trichoderma* spp. strains. Arrows represent the genes, and their size is proportional to the length of the corresponding genomic region. The GT-BG are *gliZ* (Z), zinc finger transcription factor; *gliI* (I), 1-aminocyclopropane-1-carboxylate synthase; *gliJ* (J), a dipeptidase; *gliP* (P), a two-module non-ribosomal peptide synthetase (NRPS); *gliC* (C), *gliF* (F), two cytochrome P450 monooxygenases; gliF* (F*), second putative ortholog; *gliM* (M), *O*-methyltransferase; *gliG* (G), a glutathione-*S*-transferase; *gliK* (K), a cyclotransferase (GGCT); *gliA* (A) or gliA* (A*), respectively, major facilitator superfamily (MFS) or ATP-binding cassette (ABC) transporter; *gliN* (N), a methyltransferase; *gliT* (T), a GT oxidase; *gliH* (H), coding a conserved hypothetical protein. The scaffolds containing the GT-BG are separated by double lines at their extremities; a single line indicates that the genes are present in the same scaffold but not clustered.

Overall, TvGv29-8 and Af293 showed a *gli-*gene-related protein sequence identity between 44 and 68% (Table 2). Thus, in addition to the cluster of eight *gli* genes, a duplet of two *gli* genes, two single *gli* genes, situated in different locations in the genome, and two related genes were identified (Figure 1).

When a similar to GT-BG and related genes search was performed in TvFT-333, using the TvGv29-8 gene sequences, a striking nucleotide identity (100%) (Supplementary Table S3A) was found with the corresponding 13 *Tvgli* genes (Supplementary Table S4). In addition, TvFT-333 showed an identical GT-BG architecture with a cluster of the same eight *gli* genes with the same order and orientation (on scaffolds 0430–0433) and two single *gli* genes, *gliA* and *gliJ*, situated respectively, in scaffolds 0040 and 0450. Differently, of the duplet of two *gli* genes, only *TvgliT* was present on scaffold 0048, while *TvgliH* was absent from the TvFT-333 genome. This result was confirmed also by blasting the TvGliH and AfGliH sequences.

To verify the distribution of the GT-BG in different loci, the sequences of the flanking regions of the gli cluster, the duplet and the single genes were compared in the two *T. virens* strains. Actually, in TvGv29-8, the gli cluster, the *gliH-gliT* duplet, and one end of *gliJ* are without flanking sequences (Figure 1). Differently, the sequences flanking *gliA* and the other end of *gliJ* confirmed their position outside of the gli cluster.

The third *T. virens* genome present in NCBI belongs to the “P” strain, IMI 30406, which does not contain any *gli* genes (Sherkhane et al., 2017); thus, it was not included in this study.

### *In silico* Identification of the GT Biosynthesis Genes and Related Genes in *Trichoderma* spp.

Putative gli clusters were successfully identified in seven strains of four different species: TaT6776, ThB97, ThTR274, Th226.95, TrRUTC30, TrQM6a, and TlSMF2 (Table 2 and Supplementary Tables S1,S4). GT-BG of some representative strains are depicted in Figure 1, where the multiple genes shared between *Trichoderma* species are shown. Interestingly, the gli cluster was not present in the TgT6085 genome. The organization of the GT-BG in these *Trichoderma* species differed considerably from *T. virens*, that is, the GT-BG were present in a single cluster with a very similar architecture: length ranging from 33,140 to 35,284 bp, presence of 11 *gli* genes, and absence of *gliH* and *gli*Z (Supplementary Table S5). Notably, the *T. reesei* GT-BG were located in a single cluster with 11 out of the 12 genes reported by Patron et al. (2007).

Table 2 and Supplementary Table S4 show the highest identities found in protein sequences using BLASTP search. The predicted products of the *gli* genes were similar to those found in *A. fumigatus* showing amino acid identities ranging from 23 to 82%.

Moreover, a very high (99.97%) intraspecies nucleotide identity of the gli clusters was found in *T. harzianum* and *T. reesei*, while interspecies identity varied from 70% between *Tagli*/*Trgli* and *Tagli*/*Tlgli*, to 88% between *Tagli*/*Thgli*. In these species, one major facilitator superfamily (MFS) transporter highly similar to *AfgliA* was located outside of the clusters, while an ATP-binding cassette (ABC) transporter was located in the gli clusters and coding for proteins with a low amino acid sequence identity to AfGliA and TvGliA (Supplementary Table S3B). These ABC transporters (*gliA*^∗^) showed very high interspecies identity (70–88%). The MFS and ABC transporter genes were found to belong to two different orthology groups. Lastly, *gliZ* relics were found in a genomic position located close to *gliG* and a second putative *gliF*, which resulted not to be an *AfgliF* ortholog. All the GT-BG identified in these species were indicated as orthologs of the corresponding *Afgli* genes by OrthoDB.

*GtmA* and *gipA* were present in all the *Trichoderma* species tested (Table 2 and Supplementary Table S4). Notably, orthologs of *TvgtmA* (TGAM01_v208885) and of *AfgipA* and *TvgipA* (TGAM01_v202426) were also found in TgT6085, where the GT-BP was absent.

### Gene Expression

**FIGURE 2:**
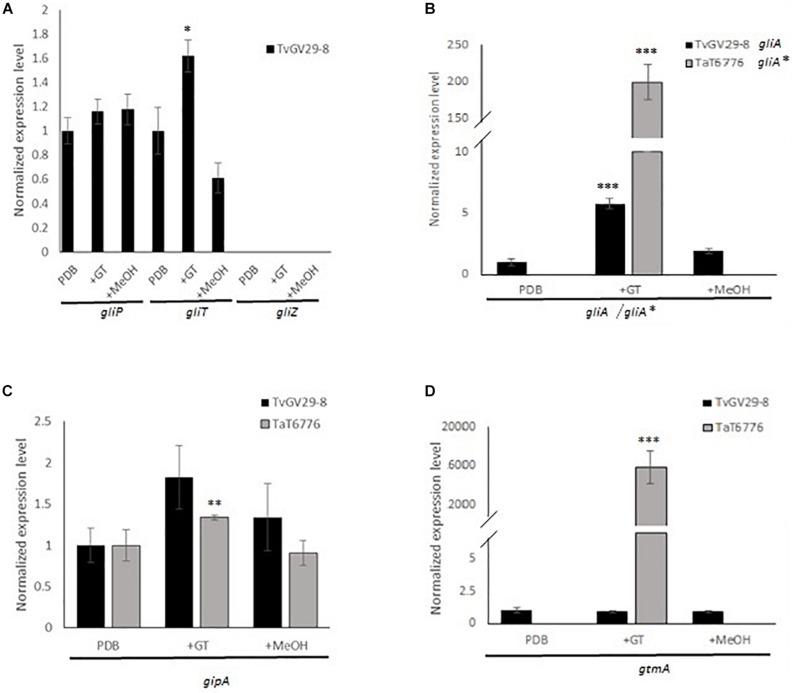
Compared gene expression analyses in TvGv29-8 and TaT6776 grown in potato dextrose broth (PDB), in PDB amended with 5 μg/ml of gliotoxin (+GT), or amended with methanol (+MeOH). **(A)** *gliP*, *gliT*, and *gliZ* in TvGv29-8, no transcriptions were detected in TaT6776; **(B)** gliA/gliA* [major facilitator superfamily (MFS)/ATP-binding cassette (ABC) transporter]; **(C)** *gipA*; **(D)** *gtmA*. Each real-time PCR (RT-PCR) reaction was performed in three biological and technical replicates. For statistical analysis, ANOVA test was applied with significance defined as a *p* value (*p ≤ 0.02; **p ≤ 0.001, ***p ≤ 0.0001).

The expression levels of *gliP* (non-ribosomal peptide synthetase), *gliT* (putative oxidoreductase), *gliA* (putative gliotoxin transporter), *gliZ* (putative Zn_2_Cys_6_ transcription factor), *gliH* (hypothetical protein), *gipA* (putative C_2_H_2_ transcription factor), and *gtmA* (putative GT *bis-*thiomethyltransferase) were analyzed (Figure 2). The results were quite different between the two *Trichoderma* strains. In the GT-producing TvGv29-8, *TvgliP*, the key gene in GT synthesis, as well as *TvgliA*, *TvgipA*, and *TvgtmA* were expressed. Among the previously identified *gliT* orthologs, only *gliT* (TRIVIDRAFT_138628) was expressed during GT production, establishing it as the putative oxidoreductase *TvgliT*. Otherwise, *TvgliZ* is still to be identified since neither the previously indicated ortholog nor the other hits revealed by BLAST analyses (data not shown) were expressed. Interestingly, *gliH* that was retrieved only in the TvGv29-8 genome was expressed in GT permissive conditions (data not shown). In TaT6776, only *gipA* was transcribed. The *TvgliH* primers were also tested in TaT6776, but no amplifications were observed.

### Expression of GT Biosynthesis Genes in the Presence of Exogenous GT

In *A. fumigatus*, *AfgliP*, *AfgliT*, *AfgliA*, and *AfgipA* are involved both in GT production and self-protection against GT toxicity (Schrettl et al., 2010; Dolan et al., 2015), while *AfgtmA* indirectly and negatively regulates the production of GT (Dolan et al., 2017), which is known to be involved in a positive regulation of its own synthesis (Cramer et al., 2006). To evaluate the role of exogenous GT on *gliP*, *gliT*, *gliA*, *gipA*, and *gtmA* expression, RT-qPCR results were compared between TvGv29-8 and TaT6776 grown in different conditions (PDB, 5 μg/ml GT, and methanol), and the significance was evaluated by ANOVA (Figure 2).

In detail, in TvGv29-8, *TvgliP* and *TvgtmA* expression levels were not significantly different in all the tested conditions. In contrast, *TvgliT* and *TvgliA* genes were upregulated by 1.6- and 6-fold, respectively, in the presence of exogenous GT. The expression levels of the transcription factor *gipA* were increased in both TvGv29-8 and TaT6776 in the presence of exogenous GT, but this effect was statistically significant only in TaT6776 (*p* ≤ 0.001). Strikingly, when the fungus TaT6776 was exposed to exogenous GT, the expression of the *TagliA*^∗^ and *TagtmA* genes was detectable and measured by 200- and 16,000-fold, respectively, compared to samples not exposed to GT (*p* ≤ 0.0001). The expression of the tested genes in the solvent control was comparable to that observed without exogenous GT.

### *T. afroharzianum* Is a GT-Non-producing Strain

**FIGURE 3:**
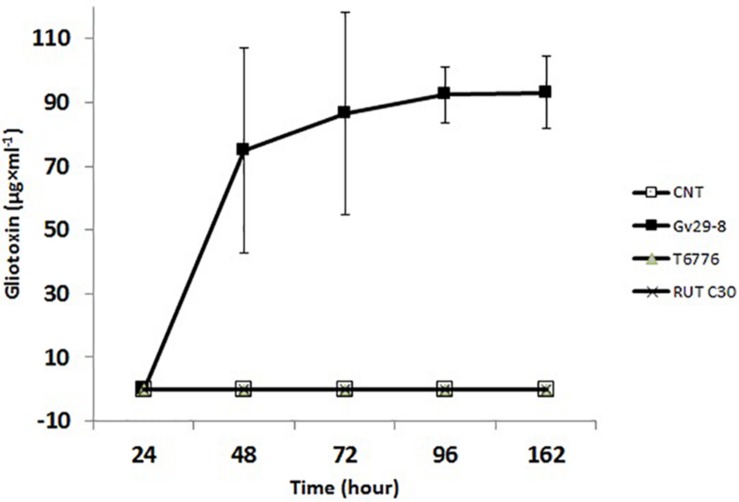
Gliotoxin time course production in TvGv29, TaT6776, and TrRUTC30, grown in Weindling medium up to 162 h, detected by high-performance liquid chromatography (HPLC). Data points represent the average ± standard deviation of biological replicate cultures (*n* = 3).

TvGv29-8 is a well-known GT-producing strain, which was confirmed in this study by HPLC analyses. The GT and BmGT concentrations referred to the level present in the original culture media. GT production began at 24 h after inoculation in Weindling medium, and it rapidly increased to a concentration of 74.9 ± 32.3 μg/ml at 48 h (Figure 3). Subsequently, the GT secreted was slowly increased to 93.1 ± 11.3 μg/ml at 162 h.

**FIGURE 4:**
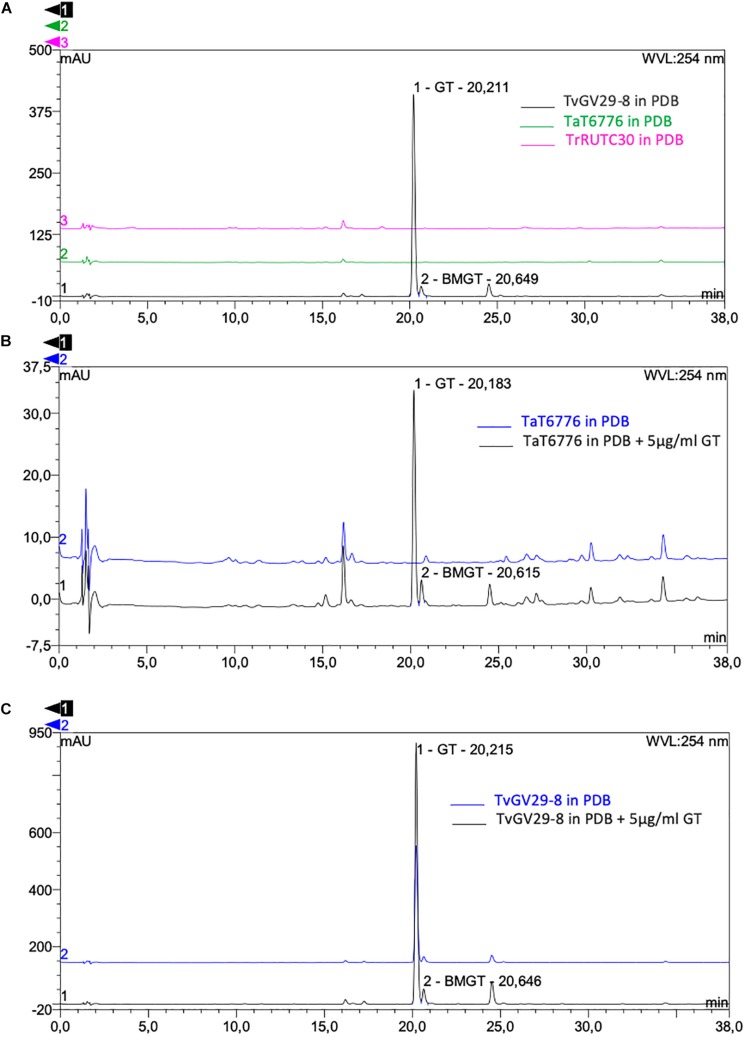
High-performance liquid chromatography (HPLC) detection of gliotoxin (GT) and *bis*(methyl)gliotoxin (BmGT) in fungal culture filtrates. **(A)** Comparison between TvGv29-8 (black), TaT6776 (green), and TrRUTC30 (magenta). The chromatograms show the presence of GT and BmGT only in TvGv29-8 sample (black). HPLC detection of gliotoxin (GT) and *bis*(methyl)gliotoxin (BmGT) in culture filtrates after 27 h incubation time of **(B)** TaT6776 and **(C)** TvGv29-8, grown in potato dextrose broth (PDB) (blue) and in PDB amended with 5 μg/ml of GT (dark gray). HPLC analysis was performed in four technical replicates.

No GT- and BmGT-related peaks were present for TaT6776 (Figure 4A), demonstrating that it does not produce GT, despite the presence of the gli cluster in its genome. No information was available on GT production by TaT6776 before this study. As expected, comparable HPLC results were found for TrRUTC30, confirming that it does not produce GT (Figure 4A).

### Exogenous Gliotoxin Induces *Bis*(Methylthio)gliotoxin Production in *Trichoderma* spp.

The production of GT and BmGT in TvGv29-8 and in TaT6776 was analyzed further after adding exogenous GT to their culture media. In this case, both GT and BmGT were present also in the culture filtrates of TaT6776 (Figure 4B). The amount of recovered GT (4.98 ± 0.9 μg/ml) corresponded to that added to the medium, while 0.19 ± 0.07 μg/ml of BmGT was produced by TaT6776.

In TvGv29-8, the increased amount of synthesized GT, from 67.09 ± 28.6 μg/ml without the addition of exogenous GT to 93.4 ± 37 μg/ml with the addition of exogenous GT, was not statistically significant (Figure 4C). Similarly, in TvGv29-8, BmGT production was observed at 27 h after inoculation at a concentration of 0.98 ± 0.4 μg/ml and 1.57 ± 0.8 μg/ml (Figure 4A), respectively, without and with the addition of exogenous GT.

### *T. afroharzianum* Is Able to Grow in the Presence of Gliotoxin

**FIGURE 5:**
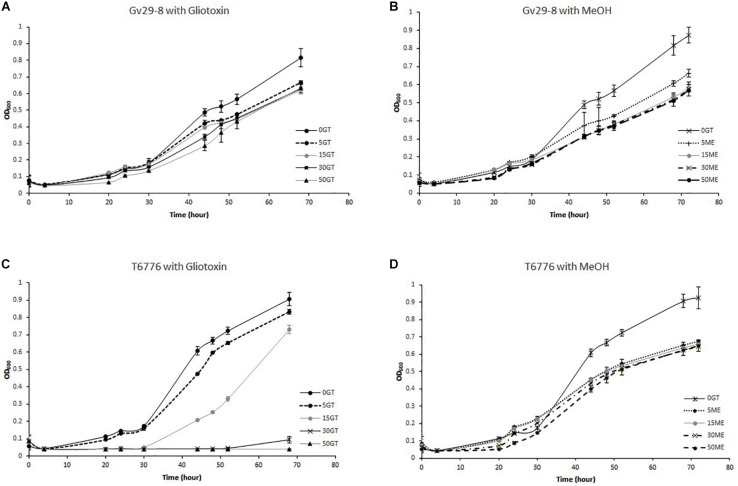
Growth of **(A)** TvGv29-8 and **(C)** TaT6776 at different concentrations of exogenous gliotoxin (0–5–15–30–50 μg/ml). Growth of **(B)** TvGv29-8 and **(D)** TaT6776 at different volumes of methanol corresponding to those present in the different gliotoxin (GT) concentrations. Data points represent the average ± standard deviation of biological replicate cultures (*n* = 3).

The ability of TvGv29-8 and TaT6776 to grow at different GT concentrations in the culture medium was verified in a multiwell assay (Figures 5A,C). The fungi differed in their sensitivity to exogenous GT. TvGv29-8 growth was only slightly affected by any GT concentration up to 50 μg/ml. In contrast, the growth of TaT6776 was completely suppressed at 50 μg/ml GT and significantly inhibited (*p* = 0.005) at 30 μg/ml GT. Nonetheless, TaT6776 did not show a dramatic sensitivity to exogenous GT, since the inhibition of its growth at 15 μg/ml GT was small (19%), although statistically significant (*p* = 0.003), compared to its growth without GT. Similarly, its growth was inhibited by 25% at 30 μg/ml GT after 96 h of incubation (data not shown). Interestingly, the presence of methanol in the media did not appear to cause a relative reduction in the growth of both TvGv29-8 and TaT6776 even at the volumes used at the highest tested GT concentrations, 30 and 50 μg/ml (Figures 5B,D).

## Discussion

Fungal SMs are defined as molecules not strictly necessary for life but critical for the lifestyle of their producers (Keller, 2019), largely conferring a better response to ecological pressures and regulated by the different environments (Bayram et al., 2008; Reverberi et al., 2010).

In *T. virens*, frequently used as biological control agent (BCA), GT is associated with its ability to control plant pathogens (Scharf et al., 2016). However, SMs are also a health concern because they represent often conserved trait associated to virulence of pathogenic fungi (Bergmann et al., 2007; Wisecaver and Rokas, 2015). An example is *A. fumigatus* where GT is considered a virulence factor (Sugui et al., 2007), and consequently, GT biosynthesis, functionality, and role have been studied in depth.

Since GT shows beneficial and deleterious functions, depending on the context, there is a need for extensive knowledge of the ability of agricultural BCAs to produce GT. To the best of our knowledge, no studies have examined the genetic potential for GT production in *T*. *afroharzianum*, *T. harzianum*, *T. longibrachiatum*, and *T. gamsii.* Moreover, the identification of the GT-BG of *T. virens* and *T. reesei* was extended and combined with preliminary insights into their functionality.

In this work, the *Trichoderma* GT-BG were searched in 6 out of the 19 species present in NCBI, for a total of 10 analyzed genomes.

Gliotoxin biosynthesis genes are quite common in *Trichoderma* spp. They are present in five species – *T. virens*, *T. harzianum*, *T. afroharzianum*, *T. reesei*, and *T. longibrachiatum* – and absent only in *T. gamsii*, among those examined in this study.

A clear polymorphism of the GT-BG structure was revealed by *in silico* analyses, and two distinct types of organization were identified. The first type was the characteristic of *T. virens* Q strains where the identification of three new putative *Tvgli* genes extended the GT-BG to 12. These genes were located at four distinct loci, and the gli cluster was separated from other essential genes (*gliA*; *gliH-gliT* duplet; *gliJ*). To assess if the GT-BG separation could be due to an incorrect genome assembly, the comparison of the two *T. virens* genomes present in NCBI was performed. Unfortunately, the absence of flanking sequences of the gli cluster and the *gliH–gliT* duplet in TvGv29-8 and the shortness of the contig length in TvFT-333 did not allow to ascertain their real position. Nonetheless, *gliJ* and *gliA* were found to be separated from the gli cluster in both strains. These findings, together with the same shared overall GT-BG structure, support the location at different loci of the GT-BG in *T. virens.*

In fungi, genes involved in SM biosynthesis are generally clustered in the genome, are coordinately regulated, and typically encode biosynthetic enzymes, one or more transcription factors, and a transporter (Nützmann et al., 2018). Nonetheless, they maintain their functionality even if located at different loci in the genome as in the trichothecene biosynthetic pathway in *Fusarium* spp. (Proctor et al., 2009). The second polymorphic type was characterized by a cluster in a single locus as described for *A. fumigatus*; it contained 11 *gli* genes and was characterized by the absence of *gliH* and by the presence of a gene coding a different class of transporter. In both GT-BG structures, the transcription factor *gliZ* was not identified.

These findings raise the questions: did *gliA*, *gliT*, *gliH*, and *gliJ* originate from the core cluster and were subsequently relocated, or did these genes originate outside the cluster and enter it? Why is *gliH* commonly absent in the *Trichoderma* genome, but so far has been found only in TvGv29-8? Does *gliZ* exist in *Trichoderma* spp.? Additional experiments are warranted to answer these questions, but were beyond the scope of this study.

The GT-BG structure polymorphism appears to be more associated with GT production than with fungal taxonomic status. In fact, while the first type was present only in *T. virens* in the GT-producing TvGv29-8, the second type was present in four species, among which two were proven unable to produce GT. The second type was also more conserved and was characterized by a high level of DNA sequence homology and by the same number, order, and orientation of the *gli* genes. Of the two *T. virens* genomes examined here, TvGv29-8 and TvFT-333, although they share an extremely high sequence identity of their GT-BG, differ in the presence/absence of *TvgliH*. Of course, these results, derived from a limited number of strains of these species, should be considered preliminary so far. However, they shed a light on a relevant fungal SM system.

Here, for the first time, the functionality of the GT-BG of *Trichoderma* spp. was analyzed using RT-qPCR developed *ad hoc* in this study. This circumscribed comparison of the GT-BG between GT-producing and GT-non-producing strains highlighted their active transcription only in TvGv29-8. The gene expression analyses combined with HPLC revealed that the key genes for GT biosynthesis are not expressed in TaT6776, which consequently fails to produce GT in these experimental conditions. The only gene expressed in default conditions in TaT6776 was *gipA*, a transcription factor, possibly involved in other biosynthetic pathways. Because metabolite production is influenced by environmental stimuli, these findings, obtained under laboratory culture conditions (i.e., on Weindling and PDB media), do not exclude the possibility of GT production on other media and under different environmental conditions. However, on the basis of the GT-BG content, the majority of the analyzed *Trichoderma* spp. strains appears unable to produce GT. A tentative explanation of this behavior could dwell on (i) the absence of *gliH*, (ii) the lack of expression of *gliT*, and (iii) possible mutation or deletion of the regulatory gene *gliZ*. In *A. fumigatus*, *gliH* and *gliT* are considered to be linked to biosynthetic genes, and their mutants *ΔgliH^26933^* and *ΔgliT^26933^* are unable to support production of GT (Schrettl et al., 2010). In *Trichoderma* spp., *gliH* is present only in the GT-producing TvGv29-8 strain and, furthermore, is located in duplet with the expressed *TvgliT*. On the basis of this work, it is possible to hypothesize that the lack of *gliH* and/or the absence of transcription of *gliT* could determine the inability of *Trichoderma* spp. to produce GT. The lack of *gliH* in TaT6776 genome is supported by the absence of its amplification by PCR in this fungus; on the other hand, the *gliH* absence possibly due to genome sequencing incompleteness cannot be ruled out owing to the impossibility to design a proper PCR protocol. Conversely, *gliH* was expressed in TvGv29-8 in GT permissive conditions.

As *Trichoderma* spp. have a comparable genetic machinery to *A. fumigatus*, a similar function of the GT biological system is hypothesized. *A. fumigatus* evades the harmful effects of GT by the combined activity of GliT and GliA in the so-called self-protection mechanism (Wang et al., 2014; Dolan et al., 2015). Upon exposure of TvGv29-8 to exogenous GT, *Tv*g*liT* and *TvgliA* were overexpressed, suggesting a possible role of the encoded proteins in the protection from the GT deleterious effects. Strikingly, exposure of the GT-non-producing TaT6776 strain to exogenous GT strongly induced *TagliA*^∗^ expression but not that of *TagliT*. In *A. fumigatus*, the gli cluster is coordinately regulated by *AfgliZ*, but some *gli* genes show also different regulation. *AfgliT*, for example, is regulated either coordinately or in an independent manner by *AfgliZ* and a still unknown regulator (Schrettl et al., 2010), and *AfgliA* is under the interdependent control of *AfgliZ* and *AfgipA* (Schoberle et al., 2014). The expression of *TagliA*^∗^ and the contextual overexpression of *TagipA*, induced by exogenous GT, could indicate a similar regulatory mechanism. These observations corroborate the mounting evidence that SM genes can also be individually expressed in response to specific stimuli. Furthermore, the expression of *TagliA*^∗^ and *TagipA* could indicate that, in GT-non-producing fungi, *gliA*, and not *gliT*, is the key gene for self-protection.

Exposure of both fungi to exogenous GT resulted also in the production and secretion of the less toxic bisdethiobis(methylthio)gliotoxin (BmGT). In *A. fumigatus*, GT is thiomethylated by the methyltransferase GtmA that acts as a post-biosynthetic negative regulator of GT biosynthesis (Dolan et al., 2014). Moreover, GtmA is known to confer resistance to GT via BmGT formation in systems where the *gliT*-mediated self-protection does not occur as in yeasts (Smith et al., 2016) and *A. niger* (Manzanares-Miralles et al., 2016). In TaT6776 exposed to the toxin, *gtmA* was functional. Thus, in *Trichoderma* GT-non-producing strains, the negative regulatory system represented by *gtmA* could have instead a role in GT self-protection. Indeed, the maintenance of an active GT self-protection system, based on the action of both *gliA* and *gtmA*, could confer them a competitive advantage providing efficient protection against exogenous GT. Actually, as shown by the multiwell growth assay, TaT6776 was able to withstand high concentrations of GT (up to 30 μg/ml), in contrast to other fungi either lacking the gli cluster or without a *gliT* ortholog (Carberry et al., 2012). As *T. gamsii* also possesses the same set of related genes but not the GT-BG, it would be interesting to test its ability to self-protect from exogenous GT. The absence or scarce induction of some TvGv29-8 *gli* genes and of *gtmA* expression by exogenous GT could reflect the high level of tolerance of this fungus to GT as shown by the growth assay or too permissive conditions.

In conclusion, the *Trichoderma* GT biological system includes two related genes, the transcription factor *gipA* and the indirect negative regulator *gtmA*, in addition to the *gli* biosynthetic genes (either clustered or sparse) similar to *A. fumigatus.* Moreover, the GT-BG are very common and polymorphic. On the basis of this circumscribed *Trichoderma* spp. screening, the most recurrent type is tentatively associated to a GT-non-producing phenotype. TaT6776, ascertained as a GT-non-producing strain with silent GT-BG, activates a *gliA^∗^-* and *gtmA*-mediated self-protection system leading to the synthesis of the less toxic metabolite BmGT.

These findings provide new insights for a safer selection of future BCAs among *Trichoderma* species, allowing to easily discard those few strains that are able to synthesize GT.

## Data Availability Statement

All datasets generated for this study are included in the article/Supplementary Material.

## Author Contributions

DB performed real-time-PCR in the presence of exogenous GT, elaborated the real-time PCR data, and drafted the manuscript. LF was involved in the designing of the whole study, and designed and validated the real-time PCR assay and multiwell growth assay. AG set up the HPLC analyses and elaborated the quantification of GT and BmGT in the analyzed samples. MB performed the HPLC analyses. EG designed the study, coordinated the work, performed the genomic analyses of the GT-BG and related genes, and contributed to draft the manuscript. All authors read and approved the final manuscript.

## Conflict of Interest

The authors declare that the research was conducted in the absence of any commercial or financial relationships that could be construed as a potential conflict of interest.
