# Supplementary material for: Enlightening Gliotoxin Biological System in Agriculturally Relevant Trichoderma spp
Source: Front Microbiol. 2020 Mar 12;11:200. doi: 10.3389/fmicb.2020.00200 (PMC7080844; doi:10.3389/fmicb.2020.00200)
Supplement: Supplementary file 1 [file Presentation_1.pptx]

## Slide 1
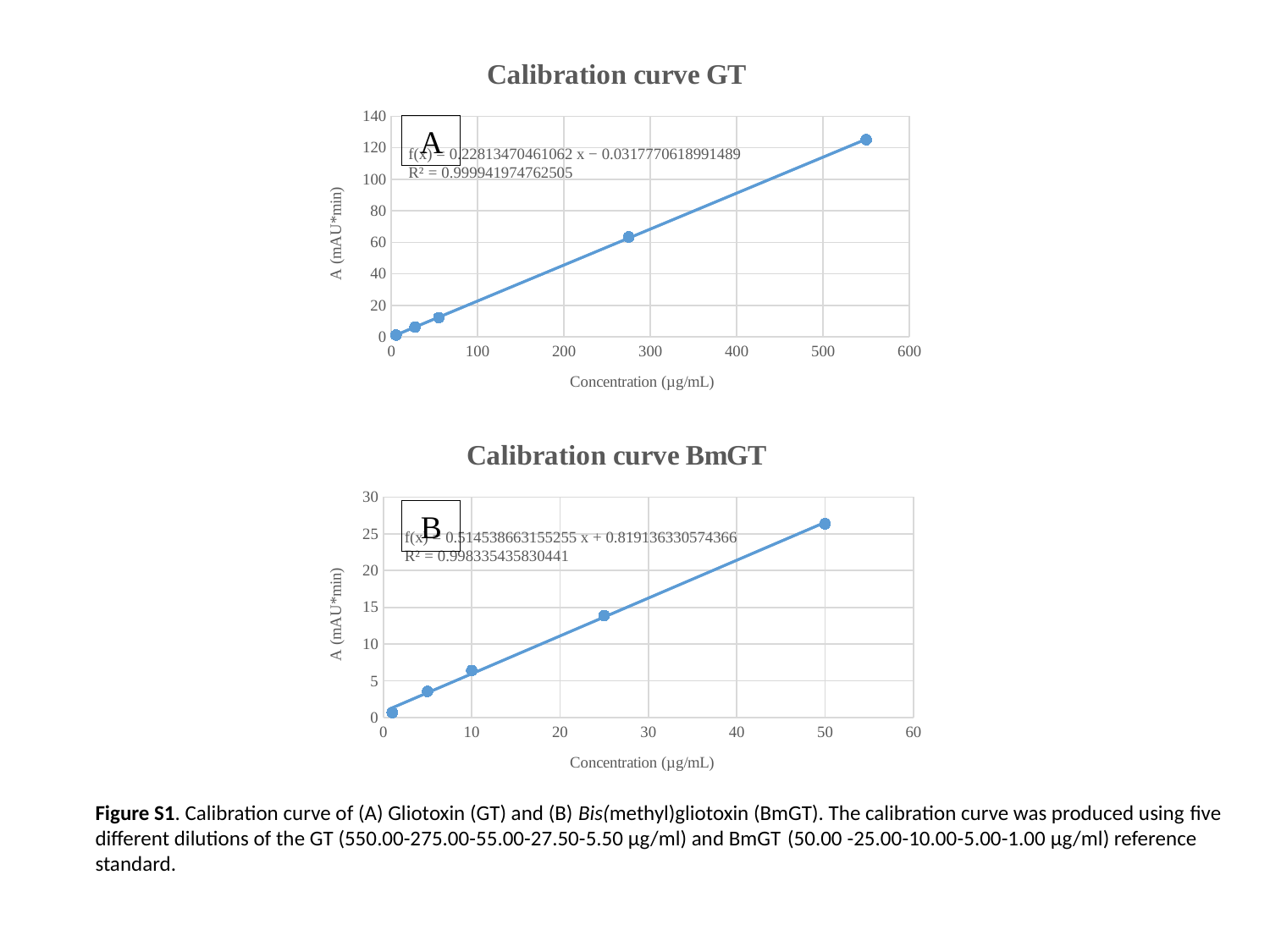

### Chart: Calibration curve GT
| Category | |
|---|---|A
### Chart: Calibration curve BmGT
| Category | |
|---|---|B
Figure S1. Calibration curve of (A) Gliotoxin (GT) and (B) Bis(methyl)gliotoxin (BmGT). The calibration curve was produced using five different dilutions of the GT (550.00-275.00-55.00-27.50-5.50 μg/ml) and BmGT (50.00 -25.00-10.00-5.00-1.00 μg/ml) reference standard.
